# Biomarkers of bone metabolism in [^223^Ra] RaCl_2_ therapy - association with extent of disease and prediction of overall survival

**DOI:** 10.1186/s13550-024-01155-w

**Published:** 2024-10-03

**Authors:** Marie Øbro Fosbøl, Niklas Rye Jørgensen, Peter Meidahl Petersen, Andreas Kjaer, Jann Mortensen

**Affiliations:** 1 Department of Clinical Physiology and Nuclear Medicine, Copenhagen University Hospital – Rigshospitalet, Copenhagen, Denmark; 2 Cluster for Molecular Imaging, Department of Biomedical Sciences, Copenhagen University Hospital – Rigshospitalet, University of Copenhagen, Copenhagen, Denmark; 3 Department of Clinical Biochemistry, Copenhagen University Hospital – Rigshospitalet, Copenhagen, Denmark; 4 Institute of Clinical Medicine, Faculty of Health and Medical Sciences, University of Copenhagen, Copenhagen, Denmark; 5 Department of Oncology, Copenhagen University Hospital – Rigshospitalet, Copenhagen, Denmark

## Abstract

**Background:**

The alpha-emitting radionuclide therapy [^223^Ra]RaCl_2_ (Radium-223) improves overall survival (OS) and time to symptomatic skeletal event (SSE) in patients with metastatic castration-resistant prostate cancer (mCRPC). Evidence suggests that the effect of Radium-223 is partly exerted through an impact on the surrounding bone matrix. We hypothesized that bone metabolism markers (BMM) could provide predictive information regarding response to Radium-223. Accordingly, the aim of this study was to investigate changes in BMM during Radium-223 therapy and evaluate association with clinical outcome.

**Methods:**

Prospective study of BMM in patients with mCRPC receiving Radium-223. Blood samples were collected before each administration of Radium-223 and the following BMM were quantified; bone-specific alkaline phosphatase (BALP), osteocalcin, procollagen type I N-propeptide (PINP), C-terminal telopeptide of type I collagen (CTX), C-terminal cross-linking telopeptide of type I collagen generated by matrix metalloproteinases (CTX-MMP), tartrate-resistant acid phosphatase isoform 5b (TRACP5b), receptor-activated nuclear factor κB ligand (RANKL), osteoprotegerin (OPG), and sclerostin. Clinical outcomes were scintigraphic progression during/after therapy, change in bone scan index (BSI), occurrence of SSE, and OS.

**Results:**

A total of 55 mCRPC patients were included. There was a significant linear association between skeletal extent of disease and CTX-MMP, PINP, BALP, and osteocalcin. No significant association between dynamics in BSI and BMM were detected. Median OS for the cohort was 14 months (95% CI: 10.7–16.8). Baseline levels of Log2-CTX-MMP (HR = 2.15 (95%CI: 1.1–4.1)) and Log2-BALP (HR = 1.59 (95%CI: 1.1–2.1)) were associated with OS. Patients with increasing CTX-MMP during therapy had significantly shorter OS (Median OS = 4 mo. (95%CI: 2.3–5.7)) than patients with stable or decreasing CTX-MMP (Median OS = 12 mo. (95%CI: 10.1–13.9), *P* < 0.001).

**Conclusion:**

BMM are significantly associated with scintigraphic extent of skeletal disease and OS in patients with mCRPC. Particularly, the bone resorption marker CTX-MMP is a promising surrogate marker for prediction of outcome in patients receiving Radium-223 therapy and could potentially improve selection of patients for therapy and assessment of response.

**Trial registration:**

Clinicaltrials.gov, NCT03247010. Registered 10th of August 2017, https://clinicaltrials.gov/study/NCT03247010?term=NCT03247010&rank=1.

**Supplementary Information:**

The online version contains supplementary material available at 10.1186/s13550-024-01155-w.

## Introduction

**Fig. 1:**
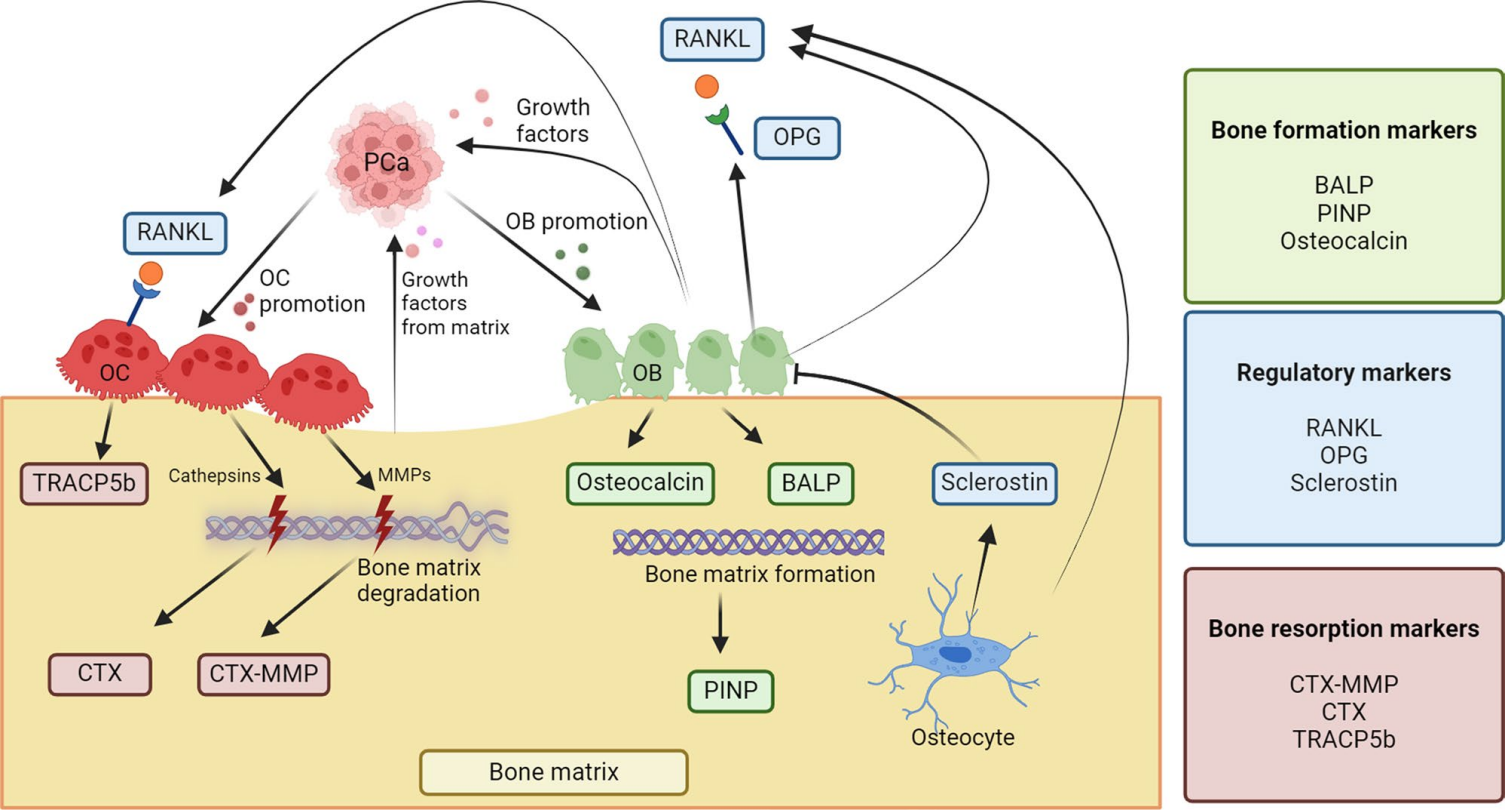
Simplified schematic representation of the bone microenvironment and the origin of biomarkers in this study. Markers of bone resorption include C-terminal telopeptide of type I collagen (CTX) and C-terminal cross-linking telopeptide of type I collagen generated by matrix metalloproteinases (CTX-MMP), which are both indicative of collagen type-I degradation through different enzymatic pathways [20]. Tartrate-resistant acid phosphatase type 5b (TRACP5b) secreted by osteoclasts (OC) correlates with the number of OCs and the degree of bone resorption [21]. Receptor-activated nuclear factor κB ligand (RANKL) plays a crucial role in bone remodelling by promoting the maturation of precursor cells into mature OCs and thereby enhancing bone resorption [22]. On the other hand, osteoprotegerin (OPG), produced by osteoblasts (OB), acts as a decoy receptor for RANKL, thereby inhibiting the binding of RANKL to its receptor RANK on OCs thereby reducing the activation of OCs. Osteocytes embedded in bone matrix produce sclerostin, which inhibits the activity of OBs and increases expression of RANKL [23–25]. Formation markers include the OB produced enzyme bone-specific alkaline phosphatase (BALP) which is involved in mineralization of bone tissue. Procollagen type I N-propeptide (PINP) is cleaved in the synthesis of collagen and the serum concentration correlates with bone formation [26]. Osteocalcin influencing bone matrix mineralization is primarily produced by OBs, but evidence suggests that the protein may also be expressed by prostate cancer cells [27, 28]. Figure created in Biorender.com/q98x774

In the advanced stages of prostate cancer (PCa) up to 90% of patients will have bone metastases leading to increased risk of pathological fracture, spinal cord compression, debilitating pain and reduced quality of life [1, 2]. The alpha-emitting radiopharmaceutical [^223^Ra]RaCl_2_ (Radium-223) selectively targets osteoblastic lesions, delivering localized radiation therapy to the bone microenvironment [3, 4]. Therapy with Radium-223 significantly improves overall survival (OS), reduces the risk of symptomatic skeletal events (SSE) and improves quality of life in metastatic castration-resistant prostate cancer (mCRPC) patients [5–7]. Development of bone metastases is dependent on interaction with the bone microenvironment, where a vicious cycle of cancer-induced increased bone remodelling causes release of growth factors promoting tumor progression (Fig. 1) [8, 9].

Evidence suggests that the anti-tumor effect of Radium-223 is partly exerted through interference with this cycle by targeting the cells of bone remodelling; osteoblasts and osteoclasts [10, 11]. Significant declines in serum concentrations of the bone formation marker alkaline phosphatase (ALP) have been reported in patients during treatment with Radium-223 [12–18]. Additionally, several studies, including a post-hoc analysis of the phase 3 ALSYMPCA trial found declines in serum ALP to be associated with better survival outcome of treatment [15, 16, 18].

Bone resorption markers are less frequently studied in the context of PCa and Radium-223 therapy. In a randomized, placebo-controlled phase II study, Nilsson et al. assessed changes in C-terminal telopeptide of type I collagen (CTX) and C-terminal cross-linking telopeptide of type I collagen generated by matrix metalloproteinases (CTX-MMP – which by former nomenclature was termed ICTP) [12]. Compared to the placebo group, patients receiving Radium-223 had a significant reduction in CTX (median − 31.4% decrease for Radium-223 vs. 31.7% increase in the placebo cohort), while median CTX-MMP increased in both groups (Radium-223: 14.6% increase vs. placebo 43.2% increase). Whether the baseline levels of bone metabolism markers (BMM) or dynamics during therapy were associated with clinical outcomes was not reported.

Agarwal et al. evaluated BMM, including bone resorption markers N-terminal telopeptide (NTX), CTX-MMP and pyridinoline (PYR) as surrogate response markers in 35 patients receiving concomitant Radium-223 and enzalutamide. Results showed changes in bone resorption markers were associated with disease control rate, while markers of bone formation (BALP and PINP) were significantly associated with prostate specific antigen (PSA) response and radiographic progression free survival (rPFS) [17]. Whether these results can be transferred to patients receiving Radium-223 as monotherapy is not elucidated.

A prospective, multicentre trial assessed baseline levels of bone resorption markers NTX and PYR and formation markers BALP and C-terminal type-I collagen propeptide (PICP) in 169 mCRPC patients initiating Radium-223 therapy as predictors of survival. Elevated baseline levels of three or more BMM were associated with poor overall survival (OS). No significant effect on OS was found for individually elevated bone resorption markers [19]. Serial measurements of BMM were not included in the study.

There is an unmet clinical need for better markers of response to Radium-223 therapy. Dynamics of PSA, which in other clinical scenarios of PCa reflects disease burden and response to therapy, does not seem to be associated with outcome of Radium-223 [15]. Assessment of BMM could provide valuable insights into the dynamic processes of bone remodelling, reflecting the activity and burden of bone metastases during the course of Radium-223 therapy. In the clinical setting, BMM could potentially add valuable predictive information, aid in treatment decision-making and patient management.

The aim of this prospective trial was to investigate dynamics of BMM in mCRPC patients during the course of Radium-223 therapy. Additionally, to explore associations between BMM levels and extent of skeletal disease, progression during therapy, OS, and occurrence of SSE, respectively. A wide panel of BMM reflecting both bone formation and –resorption, as well as factors regulating bone turnover were included in the analysis.

## Methods

### Study design

Prospective, single-centre study of patients with mCRPC referred for Radium-223 therapy at our institution from August 2017 to May 2019. Inclusion criteria were mCRPC and eligibility for Radium-223 therapy. Patients were considered eligible for therapy if they had mCRPC with symptomatic bone metastases, ECOG performance score ≤ 2, no visceral metastases or lymph node metastases > 3 cm diameter. From July 2018 new national criteria for Radium-223 therapy were implemented stating, in addition to the abovementioned criteria, patients were only eligible if they had received ≥ 2 other systemic therapies for mCRPC and no other treatment options were available. Exclusion criteria for the study included inability to understand the study protocol and therefore to give informed consent.

Radium-223 therapy was administered according to manufacturer’s guidelines as 55 kBq/kg intravenously every four weeks for up to six cycles. Routine baseline imaging consisted of planar whole-body bone scintigraphy and computed tomography (CT) of thorax and abdomen. Bone scintigraphy was repeated after three cycles and after end of therapy (EOT). Bone scintigraphies available in Digital Imaging and Communications in Medicine (DICOM) format were additionally analysed using Exini aBSI (EXINI Diagnostics, Lund, Sweden) to obtain Bone scan index (BSI). Least significant change in BSI was defined as an increase/decrease of ≥ 0.3.

Concomitant therapy with other bone supportive therapy (BST) i.e., bisphosphonates or denosumab was administered according to clinical guidelines. No concomitant treatment with abiraterone, enzalutamide or chemotherapy was undertaken.

Clinical information was collected from hospital records 18 months from inclusion or until date of death from any cause. Records were assessed for radiographic progression according to PCWG2 criteria and occurrence of symptomatic skeletal event (pathological fracture, spinal cord compression, required external beam radiation therapy or orthopaedic intervention due to metastatic complications).

The study protocol was approved by the Ethical Committee of the Capital Region of Denmark (protocol no. H-16031883). Written informed consent was obtained from all patients. The study was registered at ClinicalTrials.gov (NCT03247010).

### Markers of bone metabolism

Blood samples were collected at baseline and in each treatment cycle at the day of Radium-223 therapy before administration of the therapy. The patients were not instructed in any dietary restrictions prior to sampling, which was performed at approximately 10 AM (+/- 1 h) to reduce the circadian variation. Blood samples were centrifuged (2000 g, 10 min) within one hour. For each assay, the sample aliquots were kept frozen at – 80 degrees Celsius until the day of analysis (up to 24 months). None of the samples had previously been thawed, and all analyses were performed immediately after thawing the samples. All samples were analysed using one single batch of each assay. Assay performance was verified using the manufacturers’ control specimens and/or own internal controls based on patient pools.

CTX was measured by the IDS-iSYS CTX (CrossLaps^®^) (Immunodiagnostic Systems, plc, Tyne and Wear, UK) on the iSYS automatic analyzer. PINP was measured by the IDS-iSYS intact PINP (Immunodiagnostic Systems) assay. Osteocalcin and TRACP5b were measured using the N-Mid Osteocalcin assay and the BoneTRAP^®^ assay, respectively (Immunodiagnostic Systems). All assays were carried out on their respective dedicated automated analyzers according to the manufacturers’ instructions. All assays are chemiluminescence immunoassays.

Sclerostin was measured using the TECOMedical Sclerostin HS EIA assay (Quidel Corporation, San Diego, CA). OPG was measured using the Biomedica Osteoprotegerin ELISA assay (Biomedica, Vienna, Austria). RANKL was measured using the Free Soluble RANKL High Sensitivity ELISA assay (Biomedica), and CTX-MMP was measured using the UniQ ICTP RIA (Aidian, Espoo, Finland). Samples for sclerostin, OPG and RANKL were analyzed in duplicate in one batch and according to the manufacturers’ instructions. Plasma was used as sample material for CTX, PINP, osteocalcin, TRACP5b, and BALP. OPG, RANKL, CTX-MMP and sclerostin were measured with serum as sample material.

Intermediary precisions listed in supplementary materialS1.

### Statistical analysis

Patient characteristics were reported by descriptive statistics and comparison of the distributions of continuous data was performed with Kruskal Wallis test. Continuous variables with a right-skewed distribution were Log2-transformed. The effect of concomitant bone supportive therapy on dynamics of bone metabolic markers was investigated using linear mixed models. Associations between BMM and BSI were evaluated by linear regression.

Survival analysis of time to progression and OS was performed using cox proportional hazards regression analysis. Kaplan Meier plots and Log Rank test was applied in cases of categorical or binary independent variables in survival analysis. In analyses of OS including baseline values as independent variables, survival time was defined as months from first Radium-223 to date of death of any cause. In analyses which included dynamics of independent variables during the course of therapy, survival time was defined as the time from last Radium-223 to date of death. Binary logistic regression analysis was used to assess associations with binary outcomes (occurrence of symptomatic skeletal event).

In case of biomarker values recorded as below detection limit a fixed value of “lower detection limit/2” was included in the calculations. Least significant change (LSC) in BMM was defined as a decrease/increase of ± 30% from baseline. Evidence regarding BMM levels and biological variation in mCRPC patients is scarce, and consequently the selected LSC was based on recommendations from other clinical settings [29].

A P-value of less than 0.05 was considered statistically significant. As the study is considered exploratory, no corrections for multiple comparisons were performed. Missing data was considered “missing completely at random” as no relevant bias was expected in data collection and analysis. Missing data were excluded pairwise in univariate analysis and listwise in multivariate analyses.

Statistical analyses were performed using IBM SPSS Statistics v. 25 (IBM Corp.).

## Results

A total of 62 mCRPC patients were screened for inclusion. Five patients declined to participate in the study. Two patients were included, but subsequently excluded as they had initiated BST in the course of Radium-223 therapy, which could obscure the evaluation of response in BMM. Consequently, the study population consisted of 55 patients. Analyses of TRACP5b and BALP were only available in 40 patients due to error in blood sampling. Results from analysis of CTX-MMP was excluded in one patient as a result of hyperlipidaemia.

Baseline characteristics are presented in Table 1. For three patients (5.5%) Radium-223 was first line of systemic mCRPC therapy, second line for 9 patients (16.4%), third line for 16 patients (29.1%), and fourth/fifth line for 27 patients (49.1%).

As displayed in Table 1, the baseline levels of biomarkers of bone resorption CTX-MMP, TRACP5b, and CTX differed depending on concomitant BST with the lowest values in patients receiving denosumab. The formation markers PINP and osteocalcin were also significantly affected by concomitant BST, where patients receiving bisphosphonates had the lowest median baseline values. RANKL at baseline was below lower limit of detection (LLD) in 34 patients (62%), of which 25 patients were in concurrent denosumab therapy. A total of 24 patients (44%) had CTX levels below LLD at baseline (23 patients treated with denosumab, and 1 patient received bisphosphonates).

Only 18 patients (32.7%) completed all six series of Radium-223. The reasons for discontinuation of therapy before completion were clinical deterioration in 16 patients (29.1%), radiographic progression in 13 patients (23.6%), bone marrow suppression in six patients (10.9%), and in two cases (3.6%) by patients’ request.

**Table 1:** Baseline characteristics

	All patients (*n* = 55)	No concomitant BST (*n* = 20)	Denosumab (*n* = 27)	Bisphosphonate (*n* = 8)	P-value*
Age **(years)**^**§**^	73	73	73	77	0.168
	(70-77)	(68-78)	(70-77)	(72-83)	
Previous systemic therapy ***n*(%)**					
**Docetaxel**					
**Cabacitaxel**	45 (82.1)	15 (75.0)	24 (88.9)	6 (75.0)	
**Enzalutamide**	25 (44.6)	8 (40.0)	15 (55.6)	2 (25.0)	
**Abiraterone**	30 (55.4)	12 (60.0)	16 (59.3)	2 (25.0)	
	31 (55.4)	8 (40.0)	17 (63.0)	6 (75.0)	
BSI ^**†, §**^	3.1	3.1	2.9	2.5	0.536
	(1.7-6.3)	(1.9-9.7)	(2.3-6.7)	(1.1-4.4)	
PSA **(µg/l)**^**§**^	146	132	204	119	0.561
	(68-295)	(49-252)	(101-716)	(58-431)	
CTX-MMP **(ng/l)**^**§**^	9.9	12.7	8.3	10.6	0.022
	(6.8-13.5)	(7.3-19.8)	(5.7-11.5)	(8.0-14.2)	
TRACP5b **(U/liter)**^**§**^	4	13	2.4	4.6	≤0.001
	(2.0-9.3)	(6.7-24.1)	(1.8-2.5)	(3.4-5.7)	
CTX **(ng/l)**^**§**^	140	867	16.5	117	≤0.001
	(17.0-3235)	(663-2250)	(16.5-31.0)	(53.5-195)	
PINP **(μg/l)**^**§**^	144	227	125	72	0.003
	(10.4-1251)	(115-357)	(53-195)	(35-163)	
Osteocalcin **(μg/l)**^**§**^	25.5	37.6	17.1	14.2	0.001
	(4.6-120)	(24.7-63.8)	(10.0-40.0)	(9.7-19.0)	
BALP **(μg/l)**^**§**^	50	64.2	42.7	34	0.064
	(10.7-508)	(40.9-268)	(27.6-151)	(27.7-49.6)	
OPG **(pmol/l)**^**§**^	6	6	5.5	6.6	0.859
	(3.2-15.1)	(4.3-7.8)	(4.5-7.5)	(4.2-8.1)	
Sclerostin **(ng/ml)**^**§**^	1.2	1.2	1.3	1.2	0.48
	(0.6-2.8)	(0.9-1.4)	(1.0-1.7)	(0.9-1-3)	
RANKL **(pmol/l)**^**§**^	0.02	0.15	0.02	0.05	≤0.001
	(0.02-0.34)	(0.02-0.23)	(0.02-0.02)	(0.02-0.08)	

Baseline characteristics of the included patients and baseline values of bone metabolism markers. Patients are grouped by concomitant bone supportive therapy. *Kruskal-Wallis test for Log2-transformed continuous variables. ^**§**^ Median values, interquartile range (25-75%) in parentheses. ^**†**^ Baseline BSI available in 51 patients

**Fig. 2:**
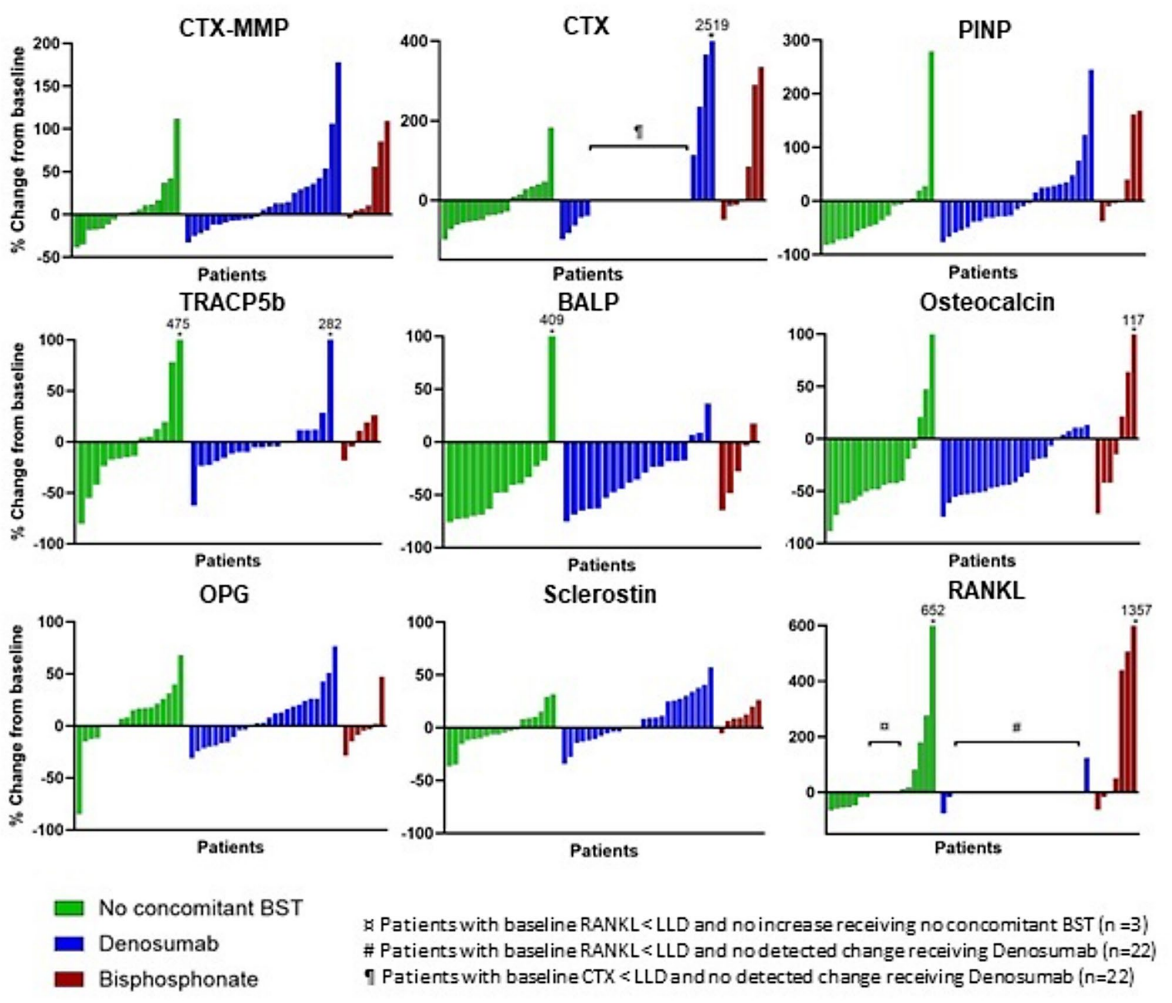
Waterfall plots of changes (%) in bone metabolism markers from baseline to end of therapy (EOT). Individual results sorted by effect size and grouped by concomitant bone supportive therapy (Green = Patients receiving no bone supportive therapy, Blue = Patients receiving denosumab, Red = Patients receiving bisphosphonate). In patients with repeated BMM measurements below lower limit of detection, no change could be recorded, as indicated in the figure. BST = Bone supportive therapy, CTX-MMP = C-terminal cross-linking telopeptide of type I collagen generated by matrix metalloproteinases, TRACP5b = Tartrate-resistant acid phosphatase type 5b, CTX = C-terminal telopeptide of type I collagen, PINP = Procollagen type I N-propeptide, BALP = Bone-specific alkaline phosphatase, OPG = Osteoprotegerin, RANKL = Receptor-activated nuclear factor κB ligand

Changes in BMM during therapy are displayed in Fig. 2. Due to consistent values below lower limit of detection changes in RANKL could not be detected in 25 patients (50%) and for CTX in 22 patients (44%). A substantial proportion of patients experienced significant declines (> 30%) in the markers of bone formation: PINP (*n* = 20, 40%), osteocalcin (*n* = 31, 62%) and BALP (*n* = 23, 61%). Regarding markers of bone resorption, the response was more diverse with a significant decline in CTX in 32% of patients, while only 6% and 11% respectively, declined in CTX-MMP and TRACP5b levels during Radium-223 therapy. The majority of patients had no significant changes in the regulatory markers; OPG (no change in 41 patients, 82%) and sclerostin (no change in 41 patients, 82%).

No significant effects of concomitant BST on dynamics of BMM during therapy on a continuous scale were detected. On a categorical scale based on LSC of 30% the proportion of patients with no significant change in RANKL (*p* = 0.002) and CTX (*p* = 0.012) were significantly increased for the patients receiving denosumab. For the other included markers, concomitant BST did not significantly affect the proportion of patients experiencing an increase or decline in BMM.

### Bone scintigraphy

Baseline bone scintigraphy was available in 51 patients, after 3 series of Radium-223 in 39 patients and a second evaluation scintigraphy in 21 patients. From baseline to end of therapy (EOT) 13 patients (33%) had declining BSI, 7 patients (18%) had stable BSI and 19 patients (49%) increased in BSI during therapy (LSC _ΔBSI_ = ± 0.3). There was no significant effect of BST status on changes in BSI.

At baseline there was a significant linear association between Log2-BSI and Log2-CTX-MMP (R^2^ = 0.25, *P* < 0.001), Log2-PINP (R^2^ = 0.46, *P* < 0.001), Log2-Osteocalcin (R^2^ = 0.19, *P* = 0.001 and Log2-BALP (R^2^ = 0.42, *P* < 0.001), respectively.

**Fig. 3:**
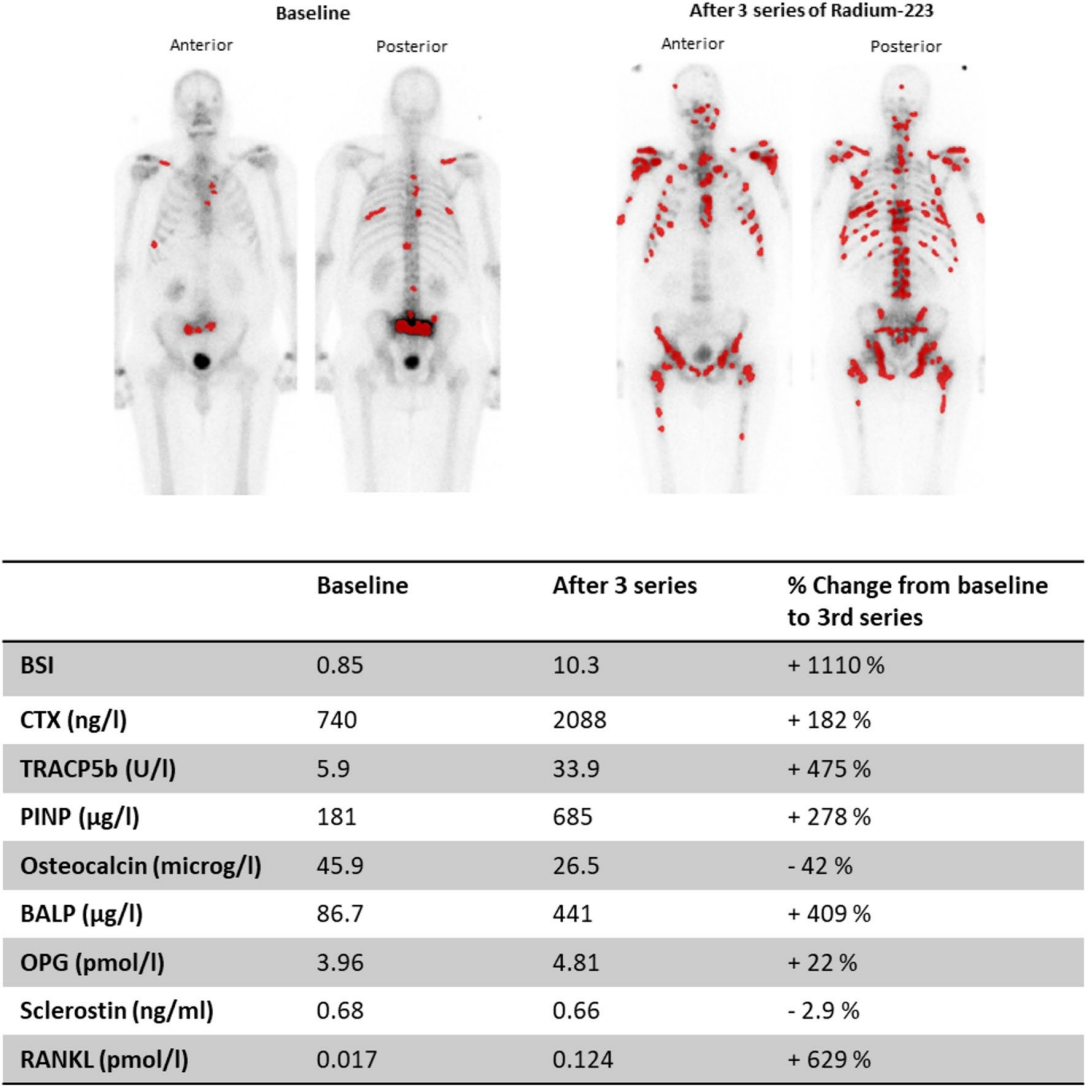
Planar bone scintigraphy (anterior/posterior) at baseline and after three series of Radium-223 from a patient who experienced a substantial increase in BSI and several biomarkers of bone metabolism during Radium-223 therapy. BSI = Bone scan index, CTX-MMP = C-terminal cross-linking telopeptide of type I collagen generated by matrix metalloproteinases, TRACP5b = Tartrate-resistant acid phosphatase type 5b, CTX = C-terminal telopeptide of type I collagen, PINP = Procollagen type I N-propeptide, BALP = Bone-specific alkaline phosphatase, OPG = Osteoprotegerin, RANKL = Receptor-activated nuclear factor κB ligand

From baseline to EOT ΔLog2-PINP (R^2^ = 0.13, *P* = 0.025) and ΔLog2-TRACP5b (R^2^ = 0.31, *P* = 0.001) had a significant linear association with ΔLog2-BSI. However, this association was assessed to be dependent on one influential outlier (Fig. 3). When excluding this case from the analysis, the associations were no longer significant.

BSI = Bone scan index, CTX = C-terminal telopeptide of type I collagen, TRACP5b = Tartrate-resistant acid phosphatase type 5b, PINP = Procollagen type I N-propeptide, BALP = Bone-specific alkaline phosphatase, OPG = Osteoprotegerin, RANKL = Receptor-activated nuclear factor κB ligand.

### Survival

Median OS was 13.7 months (95% CI: 10.7–16.8) from date of first Radium-223. In univariate Cox regression analysis of baseline values Log2-CTX-MMP, Log2-PINP, Log2-BALP, Log2-OPG, and Log2-BSI were associated with overall survival (Table 2). In multivariate analysis baseline levels of Log2-CTX-MMP (HR = 2.15 (95%CI: 1.1–4.1, *P* = 0.021)) and Log2-BALP (HR = 1.59 (95%CI: 1.1–2.1, *P* = 0.02)) were significantly associated with OS.

**Table 2:** Baseline predictors of overall survival

	Univariate analysis		*P*-value	Multivariate analysis		*P*-value
	Hazard ratio	95% CI		Hazard ratio	95% CI	
CTX-MMP (ng/l)	1.64	1.09–2.47	0.019	2.15	1.12–4.13	0.021
TRACP5 (U/liter)	1.15	0.87–1.52	0.341			
OPG (pmol/l)	2.05	1.10–3.81	0.024			
Sclerostin (ng/ml)	1.33	0.82–2.13	0.246			
BALP (µg/l)	1.39	1.05–1.85	0.021	1.51	1.07–2.13	0.018
PINP (µg/l)	1.32	1.06–1.63	0.013			
Osteocalcin (µg/l)	1.18	0.93–1.50	0.180			
CTX (ng/l)	1.03	0.93–1.13	0.588			
RANKL (pmol/l)	1.06	0.89–1.28	0.511			
BSI	1.38	1.08–1.76	0.01			
Concomitant BST* Denosumab Bisphosphonate	0.78 1.97	0.42–1.47 0.84–4.60	0.443 0.118			

Univariate and multivariate Cox proportional hazards regression analysis for baseline predictors of OS. Independent variables are Log2-transformed. * Categorical variable with “No concomitant BST” as indicator. BSI = Bone scan index, CTX-MMP = C-terminal cross-linking telopeptide of type I collagen generated by matrix metalloproteinases, TRACP5b = Tartrate-resistant acid phosphatase type 5b, CTX = C-terminal telopeptide of type I collagen, PINP = Procollagen type I N-propeptide, BALP = Bone-specific alkaline phosphatase, OPG = Osteoprotegerin, RANKL = Receptor-activated nuclear factor κB ligand, BST = Bone supportive therapy

**Fig. 4:**
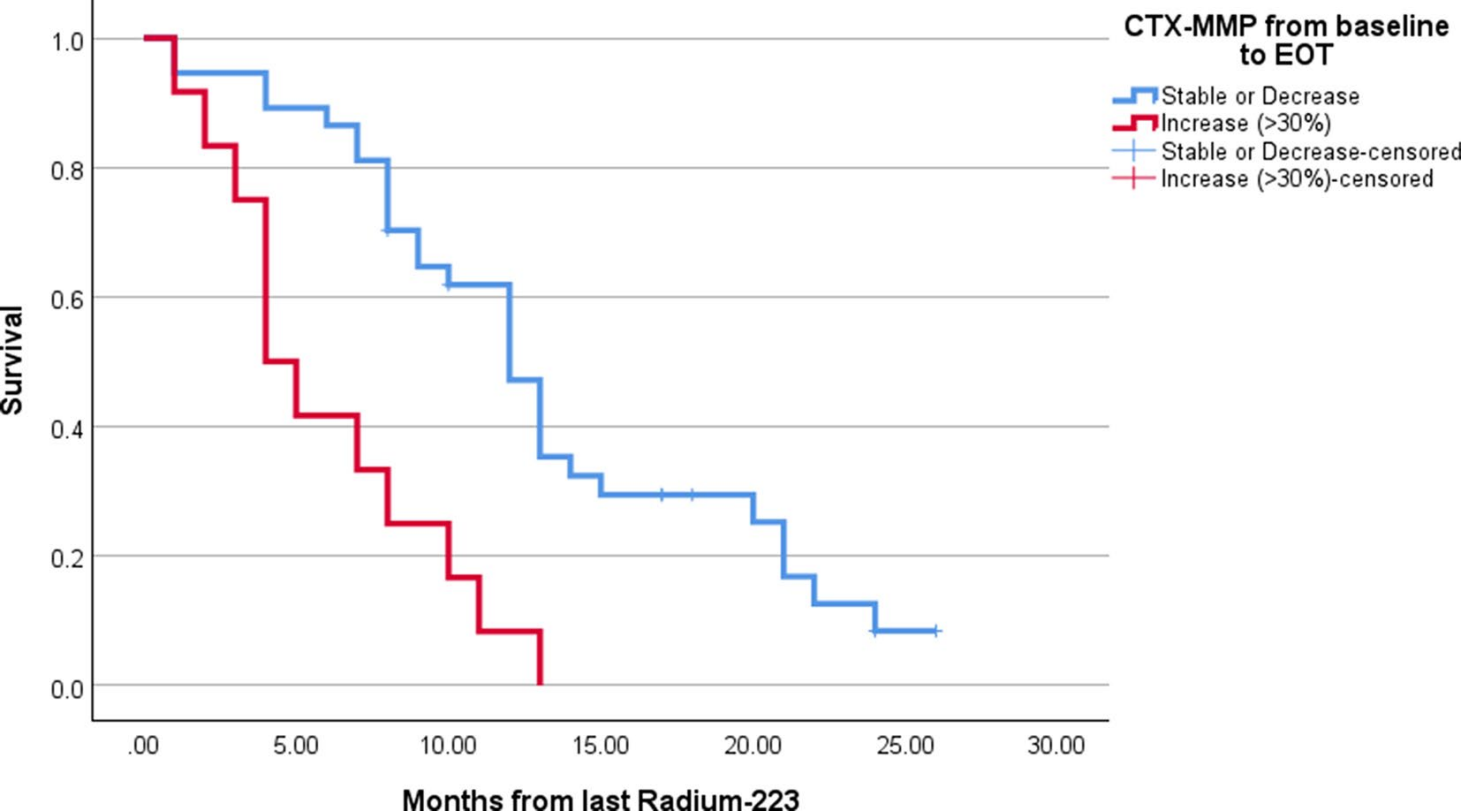
Overall survival (OS) from last series of Radium-223 dependent on changes in CTX-MMP from baseline to end-of-therapy (EOT). Patients are grouped according to whether their level of CTX-MMP was stable or decreasing level during therapy vs. increase above LSC (≥ 30%))

Increasing levels > LSC from baseline to EOT of CTX-MMP (HR = 3.8 (95%CI: 1.8–7.8, *P* < 0.001)), osteocalcin (HR = 6.0 (95%CI: 2.0-18.4, *P* = 0.002)), and BSI (HR = 2.7 (95%CI: 1.26–5.75, *P* = 0.011)) were associated with OS in univariate analysis. In multivariate analysis including concomitant BST in the regression model, only change in CTX-MMP remained a significant predictor of OS. Patients who experienced a significant increase in CTX-MMP during therapy had significantly shorter OS (Median OS = 4.0 mo. (95%CI: 2.3–5.7)) than patients with stable or decreasing CTX-MMP (Median OS = 12.0 mo. (95%CI: 10.1–13.9), *P* < 0.001) (Fig. 4).

### Symptomatic skeletal event

A total of 25 patients (45.5%) experienced a SSE during the first 12 months after initiation of Radium-223 therapy; 10 patients (18.2%) required EBRT, 10 patients (18.2%) suffered spinal cord compression, three patients (5.5%) suffered a symptomatic pathological fracture and two patients (3.6%) required orthopaedic surgery as a consequence of metastatic skeletal involvement. There was no significant association between baseline values of BMM, changes during therapy, BST status and occurrence of SSE.

## Discussion

BMM are particularly interesting in the context of Radium-223 therapy due to the bone-targeted nature of the calcium-mimetic radionuclide and the fact that response evaluation of this therapy remains a clinical challenge. This is to our knowledge the first study to analyse an extensive panel of BMM in mCRPC patients during Radium-223 therapy including markers of bone formation, bone resorption and regulatory markers. Previous studies of BMM in Radium-223 therapy have mainly focused on predictive value of baseline BMM [13, 18, 19] dynamics of bone formation markers [12, 13, 15, 16, 18] and to a lesser extent the dynamics of resorption markers during therapy [12, 14, 17].

Results from this present study showed a heterogeneous response in BMM to Radium-223 therapy. Overall, a substantial proportion of patients experienced a significant decrease in the markers of bone formation, while only a subset of patients declined in bone resorption markers. This is in line with existing evidence from a preclinical study by Suominen et al. showing a reduction in number of osteoblasts and suppressed pathological bone formation after Radium-223 in a mouse model of PCa bone metastases [11]. Additionally, the number of osteoclasts was significantly reduced after Radium-223 therapy in one type of xenograft model, indicating that Radium-223 can exert an effect on bone resorption. In accordance, our study demonstrated that the resorption marker CTX-MMP was significantly associated with OS. The level of CTX-MMP at baseline, but also more importantly the dynamics of CTX-MMP during the course of Radium-223 therapy were predictive of OS. In a multivariate Cox regression analysis patients who increased in CTX-MMP during therapy had significantly worse OS than patients with stable or declining CTX-MMP. The established disease markers ALP and BSI were included in the regression model but were not significantly associated with OS in the multivariate analysis.

At baseline, the levels of CTX-MMP, PINP, BALP, and osteocalcin showed significant linear association with BSI as a marker of skeletal disease extent. However, there was no association between the dynamics of these BMM and changes in BSI during therapy. This could be due to the inability of bone scintigraphy to detect decrease in skeletal tumor burden, as the scintigraphic lesions can persist despite successful therapy. Although bone scintigraphy currently is regarded as a cornerstone in management of mCRPC patients [30], results imply that CTX-MMP could potentially offer additional information regarding skeletal response to Radium-223.

Why CTX-MMP seems to be more closely associated with disease burden and treatment response in skeletal metastases compared to other BMM could be attributable to the origin of the marker. CTX and CTX-MMP both reflect collagen degradation, but by different enzymatic pathways, the activity of which varies according to the pathophysiology of the bone disease. In normal physiological bone remodelling, the collagenolytic effect is mainly exerted by cysteine proteinases where the degradation results in release of CTX [20, 31]. CTX levels are increased in diseases such as postmenopausal osteoporosis and decrease during antiresorptive therapy with bisphosphonates [32, 33].

In contrast, the levels of CTX-MMP which stems from collagen degradation by matrix metalloproteinases (MMP) show minor changes to antiresorptive therapy [32]. Evidence suggests that MMP play an important role in establishment and growth of PCa bone metastases [34] and clinical studies have demonstrated that high levels of CTX-MMP is associated with presence of skeletal involvement in patients with PCa [35–37]. Hence, CTX-MMP could potentially have higher specificity for assessment of bone metastases than other BMM, as it primarily reflects the pathological bone turnover.

Previous studies have found that the bone formation marker BALP at baseline was predictive of OS, which was confirmed in the present study. However, opposed to previous reports neither BALP nor the other markers of bone formation were predictive of OS when assessing dynamics during therapy. This discrepancy could potentially be explained by lack of power in the study, as only a minor subset of patients in this study experienced a significant increase in these markers, skewing the distribution between the groups.

A conceivable confounding factor in the present study is concomitant BST, which was administered to the patients at the discretion of the treating physician according to clinical guidelines. Baseline levels of CTX-MMP, TRACP5b, CTX, RANKL, osteocalcin, and PINP were dependent on whether the patients received concomitant BST as reported previously in prostate cancer patients with bone metastatic disease [38–40]. During Radium-223 therapy, patients receiving denosumab were more likely to have unchanged RANKL and CTX during therapy, compared to patients receiving no BST or bisphosphonates. However, a substantial proportion of patients receiving denosumab had baseline levels of RANKL and/or CTX below LLD, meaning that only substantial increases in these markers would be detected. For the other BMM, there was no significant effect of BST on changes during therapy, but the study may be underpowered for this analysis, especially regarding patients treated with bisphosphonate.

Another limitation of the study is the biological variability of BMM, that may obscure the validity of the serial measurements. For several BMM, a marked circadian variation in plasma levels has been reported, most prominent in the bone resorption marker CTX, in which levels can vary up to 50% across the day [41, 42]. This is particularly affected by food intake/fasting [42]. In the present study, samples were collected in the morning between from 9 AM to 11 AM to reduce the circadian variation. To minimize discomfort for the included patients, we chose not to instruct the patients in any dietary precautions prior to sampling, although this may have had an impact on the measured levels of CTX. However, the other BMM are only to a small extent affected by food intake and the results are therefore valid even when samples were not collected in the fasting state.

Despite the mentioned limitations of the study, we identified an interesting possibility of predicting response to Radium-223 therapy by assessing CTX-MMP. As a marker of an alternative bone resorption pathway associated with metastatic disease, CTX-MMP may provide a more specific assessment of skeletal involvement of PCa. In the clinical setting, CTX-MMP could potentially contribute to identify patients who are unlikely to benefit from further Radium-223 therapy, since an increase in CTX-MMP was associated with a very poor prognosis.

Nevertheless, further studies are required to validate the findings and preferably with a larger study cohort to adequately assess the consequences of concomitant BST. Another interesting perspective for future trials could be assessment of BMM in other clinical settings of mCRPC, such as radionuclide therapy targeting prostate-specific membrane antigen (PSMA).

## Conclusion

In conclusion, this study demonstrates the dynamics of BMM during Radium-223 therapy in mCRPC patients and evaluates the potential of BMM as markers of assessing response to therapy, which currently is a significant clinical challenge. Our results show, that the bone resorption marker CTX-MMP is a stronger marker of response to Radium-223 than the established biomarkers BSI and BALP. Including evaluation of CTX-MMP in the clinical management of mCRPC could potentially improve selection of patients for Radium-223 and assessment of response, especially by identifying patients who are unlikely to benefit from further therapy. However, validation studies are required to confirm the findings and further explore the significance of potential confounders in dynamics of BMM in patients receiving Radium-223.

## Electronic supplementary material

Below is the link to the electronic supplementary material.

Supplementary Material 1

## Data Availability

The datasets generated and/or analysed during the current study are not publicly available due to patient confidentiality and data safety regulations, but anonymized data are available form from the corresponding author on reasonable request.

## References

[1] Bubendorf L, Schöpfer a, Wagner U, Sauter G, Moch H, Willi N, et al. Metastatic patterns of prostate cancer: an autopsy study of 1,589 patients. Hum Pathol. 2000;31(5):578–83. 10836297 10.1053/hp.2000.6698

[2] Nørgaard M, Jensen AØ, Jacobsen JB, Cetin K, Fryzek JP, Sørensen HT. Skeletal related events, bone metastasis and survival of prostate cancer: a population based cohort study in Denmark (1999 to 2007). J Urol [Internet]. 2010 Jul [cited 2015 Nov 26];184(1):162–7. Available from: http://www.ncbi.nlm.nih.gov/pubmed/20483155 10.1016/j.juro.2010.03.034 20483155

[3] Carrasquillo Ja, O’Donoghue Ja, Pandit-Taskar N, Humm JL, Rathkopf DE, Slovin SF, et al. Phase i pharmacokinetic and biodistribution study with escalating doses of 223Ra-dichloride in men with castration-resistant metastatic prostate cancer. Eur J Nucl Med Mol Imaging. 2013;40(9):1384–93. 23653243 10.1007/s00259-013-2427-6 PMC5468165

[4] Henriksen G, Breistøl K, Bruland ØS, Fodstad Ø, Larsen RH. Significant antitumor effect from bone-seeking, α-particle-emitting 223Ra demonstrated in an experimental skeletal metastases model. Cancer Res. 2002;62(11):3120–5. 12036923

[5] Sartor O, Coleman R, Nilsson S, Heinrich D, Helle SI, O’Sullivan JM, et al. Effect of radium-223 dichloride on symptomatic skeletal events in patients with castration-resistant prostate cancer and bone metastases: results from a phase 3, double-blind, randomised trial. Lancet Oncol. 2014;15(7):738–46. 24836273 10.1016/S1470-2045(14)70183-4

[6] Parker C, Nilsson S, Heinrich D, Helle SII, O’Sullivan JMM, Fosså SDD et al. Alpha emitter radium-223 and survival in metastatic prostate cancer. N Engl J Med [Internet]. 2013 Jul 18 [cited 2016 Dec 7];369(3):213–23. Available from: http://www.ncbi.nlm.nih.gov/pubmed/23863050 10.1056/NEJMoa1213755 23863050

[7] Nilsson S, Cislo P, Sartor O, Vogelzang NJ, Coleman RE, O’Sullivan JM et al. Patient-reported quality-of-life analysis of radium-223 dichloride from the phase III ALSYMPCA study. Annals of oncology: official journal of the European Society for Medical Oncology / ESMO [Internet]. 2016 May [cited 2016 Aug 19];27(5):868–74. Available from: http://www.ncbi.nlm.nih.gov/pubmed/26912557 10.1093/annonc/mdw065 PMC4843190 26912557

[8] Furesi G, Rauner M, Hofbauer LC. Emerging Players in Prostate Cancer-Bone Niche Communication. Trends Cancer [Internet]. 2021 Feb 1 [cited 2022 Nov 7];7(2):112–21. Available from: https://pubmed.ncbi.nlm.nih.gov/33274720/10.1016/j.trecan.2020.09.006 33274720

[9] Doglioni G, Parik S, Fendt SM. Interactions in the (Pre)metastatic Niche Support Metastasis Formation. Front Oncol [Internet]. 2019 [cited 2022 Nov 7];9(MAR). Available from: https://pubmed.ncbi.nlm.nih.gov/31069166/10.3389/fonc.2019.00219 PMC6491570 31069166

[10] Abou DS, Ulmert D, Doucet M, Hobbs RF, Riddle RC, Thorek DLJ. Whole-Body and Microenvironmental Localization of Radium-223 in Naïve and Mouse Models of Prostate Cancer Metastasis. J Natl Cancer Inst [Internet]. 2016 May 18 [cited 2017 Aug 11];108(5):djv380. Available from: http://www.ncbi.nlm.nih.gov/pubmed/26683407 10.1093/jnci/djv380 PMC4849807 26683407

[11] Suominen MI, Fagerlund KM, Rissanen JP, Konkol YM, Morko JP, Peng Z et al. Radium-223 Inhibits Osseous Prostate Cancer Growth by Dual Targeting of Cancer Cells and Bone Microenvironment in Mouse Models. Clinical Cancer Research [Internet]. 2017 Aug 1 [cited 2017 Nov 10];23(15):4335–46. Available from: http://www.ncbi.nlm.nih.gov/pubmed/28364014 10.1158/1078-0432.CCR-16-2955 PMC5540794 28364014

[12] Nilsson S, Franzén L, Parker C, Tyrrell C, Blom R, Tennvall J, et al. Bone-targeted radium-223 in symptomatic, hormone-refractory prostate cancer: a randomised, multicentre, placebo-controlled phase II study. Lancet Oncol. 2007;8(7):587–94. 17544845 10.1016/S1470-2045(07)70147-X

[13] Liu RF, Juwara L, Ferrario C, Probst SM. Outcomes and Factors Associated with Completion of Radium-223 Therapy. Nucl Med Mol Imaging [Internet]. 2022 Oct 1 [cited 2023 Mar 8];56(5):228–35. Available from: https://pubmed.ncbi.nlm.nih.gov/36310835/10.1007/s13139-022-00760-8 PMC9508311 36310835

[14] Nakashima K, Makino T, Kadomoto S, Iwamoto H, Yaegashi H, Iijima M et al. Initial experience with radium-223 chloride treatment at the Kanazawa University Hospital. Anticancer Res [Internet]. 2019 May 1 [cited 2021 Jan 26];39(5):2607–14. Available from: https://pubmed.ncbi.nlm.nih.gov/31092459/10.21873/anticanres.13384 31092459

[15] Sartor O, Coleman RE, Nilsson S, Heinrich D, Helle SI, O’Sullivan JM et al. An exploratory analysis of alkaline phosphatase, lactate dehydrogenase, and prostate-specific antigen dynamics in the phase 3 ALSYMPCA trial with radium-223. Annals of Oncology [Internet]. 2017 May 1 [cited 2017 Aug 11];28(5):1090–7. Available from: http://www.ncbi.nlm.nih.gov/pubmed/28453701 10.1093/annonc/mdx044 PMC5406754 28453701

[16] Badrising SK, Louhanepessy RD, van der Noort V, Coenen JLLM, Hamberg P, Beeker A et al. A prospective observational registry evaluating clinical outcomes of Radium-223 treatment in a nonstudy population. Int J Cancer. 2019;147(4):1143–51. 10.1002/ijc.32851 PMC7383569 31875956

[17] Agarwal N, Nussenzveig R, Hahn AW, Hoffman JM, Morton K, Gupta S et al. Prospective Evaluation of Bone Metabolic Markers as Surrogate Markers of Response to Radium-223 Therapy in Metastatic Castration-resistant Prostate Cancer. Clin Cancer Res [Internet]. 2020 May 1 [cited 2020 May 12];26(9):2104–10. Available from: http://www.ncbi.nlm.nih.gov/pubmed/31937614 10.1158/1078-0432.CCR-19-2591 PMC8662311 31937614

[18] van der Doelen MJ, Stockhaus A, Ma Y, Mehra N, Yachnin J, Gerritsen WR et al. Early alkaline phosphatase dynamics as biomarker of survival in metastatic castration-resistant prostate cancer patients treated with radium-223. Eur J Nucl Med Mol Imaging [Internet]. 2021 Sep 1 [cited 2021 Sep 17];48(10):3325. Available from: https://pubmed.ncbi.nlm.nih.gov/33686456//pmc/articles/PMC8426246/. 10.1007/s00259-021-05283-6 PMC8426246 33686456

[19] Romero-Laorden N, Lorente D, de Velasco G, Lozano R, Herrera B, Puente J et al. Prospective Assessment of Bone Metabolism Biomarkers and Survival in Metastatic Castration-resistant Prostate Cancer Patients Treated with Radium-223: The PRORADIUM Study. Eur Urol Oncol [Internet]. 2024 Jun 1 [cited 2024 Jul 29];7(3):447–55. Available from: https://pubmed.ncbi.nlm.nih.gov/37838555/10.1016/j.euo.2023.09.015 37838555

[20] Garnero P, Ferreras M, Karsdal MA, Nicamhlaoibh R, Risteli J, Borel O et al. The Type I Collagen Fragments ICTP and CTX Reveal Distinct Enzymatic Pathways of Bone Collagen Degradation. Journal of Bone and Mineral Research [Internet]. 2003 May 1 [cited 2021 May 20];18(5):859–67. Available from: https://pubmed.ncbi.nlm.nih.gov/12733725/10.1359/jbmr.2003.18.5.859 12733725

[21] Zenger S, He W, Ek-Rylander B, Vassiliou D, Wedin R, Bauer H, et al. Differential expression of tartrate-resistant acid phosphatase isoforms 5a and 5b by tumor and stromal cells in human metastatic bone disease. Clin Exp Metastasis. 2011;28(1):65–73. 20967488 10.1007/s10585-010-9358-4

[22] Zhang Y, Liang J, Liu P, Wang Q, Liu L, Zhao H. The RANK/RANKL/OPG system and tumor bone metastasis: potential mechanisms and therapeutic strategies. Frontiers in Endocrinology. Volume 13. Frontiers Media S.A.; 2022. 10.3389/fendo.2022.1063815 PMC9800780 36589815

[23] Van Bezooijen RL, Roelen BAJ, Visser A, Van Der Wee-Pals L, De Wilt E, Karperien M, et al. Sclerostin is an osteocyte-expressed negative Regulator of bone formation, but not a classical BMP antagonist. J Exp Med. 2004;199(6):805–14. 15024046 10.1084/jem.20031454 PMC2212719

[24] Wijenayaka AR, Kogawa M, Lim HP, Bonewald LF, Findlay DM, Atkins GJ. Sclerostin Stimulates Osteocyte Support of Osteoclast Activity by a RANKL-Dependent Pathway. PLoS One [Internet]. 2011 Oct 4 [cited 2023 Jun 16];6(10):25900. Available from: https://pubmed.ncbi.nlm.nih.gov/21991382/10.1371/journal.pone.0025900 PMC3186800 21991382

[25] Yavropoulou MP, van Lierop AH, Hamdy NAT, Rizzoli R, Papapoulos SE. Serum sclerostin levels in Paget’s disease and prostate cancer with bone metastases with a wide range of bone turnover. Bone. 2012;51(1):153–7. 22579776 10.1016/j.bone.2012.04.016

[26] Chavassieux P, Portero-Muzy N, Roux JP, Garnero P, Chapurlat R. Are biochemical markers of bone turnover representative of bone histomorphometry in 370 postmenopausal women? J Clin Endocrinol Metab. 2015;100(12):4662–8. 26505821 10.1210/jc.2015-2957

[27] Zoch ML, Clemens TL, Riddle RC. New insights into the biology of osteocalcin. Vol. 82, Bone. Elsevier Inc.; 2016. pp. 42–9. 10.1016/j.bone.2015.05.046 PMC4670816 26055108

[28] Gardner TA, Lee SJ, Lee SD, Li X, Shirakawa T, Kwon DD, et al. Differential expression of osteocalcin during the metastatic progression of prostate cancer. Oncol Rep. 2009;21(4):903–8. 19287987 10.3892/or_00000302

[29] Wheater G, Elshahaly M, Tuck SP, Datta HK, van Laar JM. The clinical utility of bone marker measurements in osteoporosis. 11, J Translational Med. 2013. 10.1186/1479-5876-11-201 PMC3765909 23984630

[30] European association of urology, European Association of Nuclear Medicine. European Society of Urogenital Radiology, European Society for Radiotherapy & Oncology, International Society of Urological Pathology, International Society of Geriatric Oncology. EAU Prostate Cancer Guidelines. Edn. presented at the EAU Annual Congress Milan 2023 [Internet]. 2023 [cited 2023 Aug 9]. Available from: https://uroweb.org/guidelines/prostate-cancer

[31] Dai R, Wu Z, Chu HY, Lu J, Lyu A, Liu J et al. Cathepsin K: The Action in and Beyond Bone. Front Cell Dev Biol [Internet]. 2020 Jun 4 [cited 2024 Aug 12];8. Available from: https://pubmed.ncbi.nlm.nih.gov/32582709/10.3389/fcell.2020.00433 PMC7287012 32582709

[32] Garnero P, Shih WJ, Gineyts E, Karpf DB, Delmas PD. Comparison of new biochemical markers of bone turnover in late postmenopausal osteoporotic women in response to alendronate treatment. J Clin Endocrinol Metab [Internet]. 1994 Dec [cited 2024 Aug 12];79(6):1693–700. Available from: https://pubmed.ncbi.nlm.nih.gov/7989477/10.1210/jcem.79.6.7989477 7989477

[33] Bønløkke SE, Rand MS, Haddock B, Arup S, Smith CD, Jensen JEB et al. Baseline bone turnover marker levels can predict change in bone mineral density during antiresorptive treatment in osteoporotic patients: the Copenhagen bone turnover marker study. Osteoporos Int [Internet]. 2022 Oct 1 [cited 2024 Aug 12];33(10):2155–64. Available from: https://pubmed.ncbi.nlm.nih.gov/35729342/10.1007/s00198-022-06457-0 35729342

[34] Bonfil RD, Fridman R, Mobashery S, Cher ML. Are matrix metalloproteinases relevant therapeutic targets for prostate cancer bone metastasis? Current Oncology [Internet]. 2008 [cited 2024 Aug 12];15(4):188. Available from: https://www.ncbi.nlm.nih.gov/pmc/articles/PMC2528310/10.3747/co.v15i4.216 PMC2528310 18769612

[35] Koizumi M, Yonese J, Fukui I, Ogata E. The serum level of the amino-terminal propeptide of type I procollagen is a sensitive marker for prostate cancer metastasis to bone. BJU Int. 2001;87(4):348–51. 11251528 10.1046/j.1464-410x.2001.00105.x

[36] Koga H, Naito S, Koto S, Sakamoto N, Nakashima M, Yamasaki T et al. Use of bone turnover marker, pyridinoline cross-linked carboxyterminal telopeptide of type I collagen (ICTP), in the assessment and monitoring of bone metastasis in prostate cancer. Prostate [Internet]. 1999 Mar 15 [cited 2021 Jan 28];39(1):1–7. Available from: https://pubmed.ncbi.nlm.nih.gov/10221259/10.1002/(sici)1097-0045(19990401)39:1<1::aid-pros1>3.0.co;2-x 10221259

[37] Kylmaälä T, Tammelal TLJ, Risteli L, Risteli J, Kontturi M, Elomaa I. Type I collagen degradation product (ICTP) gives information about the nature of bone metastases and has prognostic value in prostate cancer. Br J Cancer [Internet]. 1995 [cited 2024 Aug 12];71(5):1061–4. Available from: https://pubmed.ncbi.nlm.nih.gov/7734300/10.1038/bjc.1995.204 PMC2033764 7734300

[38] Mercatali L, Ricci M, Scarpi E, Serra P, Fabbri F, Ricci R, et al. RANK/RANK-L/OPG in patients with bone metastases treated with anticancer agents and zoledronic acid: a prospective study. Int J Mol Sci. 2013;14(6):10683–93. 23702841 10.3390/ijms140610683 PMC3709696

[39] De La Piedra C, Alcaraz A, Bellmunt J, Meseguer C, Gómez-Caamano A, Ribal MJ, et al. Usefulness of bone turnover markers as predictors of mortality risk, disease progression and skeletal-related events appearance in patients with prostate cancer with bone metastases following treatment with zoledronic acid: TUGAMO study. Br J Cancer. 2013;108(12):2565–72. 23722472 10.1038/bjc.2013.270 PMC3694249

[40] Smith MR, Saad F, Egerdie B, Sieber P, Tammela TLJ, Leder BZ et al. Denosumab and changes in bone turnover markers during androgen deprivation therapy for prostate cancer. J Bone Miner Res [Internet]. 2011 Dec [cited 2023 Mar 6];26(12):2827–33. Available from: https://pubmed.ncbi.nlm.nih.gov/21898590/10.1002/jbmr.492 PMC3222788 21898590

[41] Diemar SS, Dahl SS, West AS, Simonsen SA, Iversen HK, Jørgensen NR. A Systematic Review of the Circadian Rhythm of Bone Markers in Blood. Calcif Tissue Int [Internet]. 2022 [cited 2022 Oct 6]; Available from: https://pubmed.ncbi.nlm.nih.gov/35305134/10.1007/s00223-022-00965-1 35305134

[42] Schini M, Vilaca T, Gossiel F, Salam S, Eastell R. Bone turnover markers: Basic Biology to Clinical Applications. Endocrine Reviews. Volume 44. Endocrine Society; 2023. pp. 417–73. 10.1210/endrev/bnac031 PMC10166271 36510335

