# Supplementary material for: Biomarkers of bone metabolism in [223Ra] RaCl2 therapy - association with extent of disease and prediction of overall survival
Source: EJNMMI Res. 2024 Oct 3;14:90. doi: 10.1186/s13550-024-01155-w (PMC11450107; doi:10.1186/s13550-024-01155-w)
Supplement: Supplementary file 1 — Supplementary Material 1 [file 13550_2024_1155_MOESM1_ESM.docx]

**Supplementary material**

*S1 – Intermediary precisions for analyses of markers of bone metabolism*

The intermediary precisions expressed as coefficients of variation for CTX were 5.3% (at CTX concentration 213 ng/L), 3.4% (869 ng/L), and 3.5% (2,113 ng/L). For PINP the intermediary precisions were 5.4% (19 µg/L), 6.5% (48 µg/L), and 6.1% (122 µg/L). For osteocalcin the intermediary precisions were 3.0% (8.73 µg/L), 3.6% (27.6 µg/L), and 3.5% (68.7 µg/L) and for TRACP5b the intermediary precision was 10.9% (3.2 U/L), 4.8% (6.2 U/L), and 5.4% (9.0 U/L). For sclerostin, the intra-assay precision was 13.3% at the 0.12 ng/mL level and 3.4% at the 1.03 ng/mL level. For OPG the intra-assay precision was 17% at 4.5 pmol/L and for RANKL the intra-assay precision was 18% at the 0.42 pmol/L level and 17% at the 0.76 pmol/L level. Finally, for CTX-MMP the intra-assay precision was 5% at both the 7.1 and 18.5 µg/L levels and 9.2% at the 25 µg/L level.
